# Supplementary material for: Monitoring of Fabric Integrity and Attrition Rate of Dual-Active Ingredient Long-Lasting Insecticidal Nets in Tanzania: A Prospective Cohort Study Nested in a Cluster Randomized Controlled Trial
Source: Insects. 2024 Feb 4;15(2):108. doi: 10.3390/insects15020108 (PMC10889750; doi:10.3390/insects15020108)
Supplement: Supplementary file 1 [file insects-15-00108-s001.zip › insects-2742211-supplementary.pdf]

Supplementary materials

Table S1: pHI categories as per WHO guideline 2013.

**Category**	**pHI value range**	**Total hole surface area in cm²**
Good	0-64	<100
Damage	65 – 642	100 – 1,000
Too torn	643+	>1,000

Table S2: Reason for attrition per net type and net age

		Interceptor® % (95%CI)	Interceptor® G2 % (95%CI)	Olyset ™ Plus % (95%CI)	Royal Guard® % (95%CI)
Thrown away	6M	11% (3 - 29)	33% (20 - 48)	69% (59 - 77)	30% (19 - 44)
	12M	31% (22 - 41)	66% (58 - 73)	66% (60 - 72)	39% (31 - 47)
	24M	64% (59 - 70)	66% (61 - 71)	87% (84 - 89)	74% (70 - 78)
	30M	73% (68 - 78)	81% (77 - 85)	86% (83 - 89)	82% (78 - 85)
	36M	79% (75 - 83)	87% (83 - 90)	90% (87 - 92)	90% (87 - 92)
Used for other purpose	6M	4% (0 - 22)	7% (2 - 20)	6% (3 - 12)	4% (0 - 14)
	12M	11% (6 - 19)	4% (2 - 9)	9% (6 - 14)	8% (5 - 14)
	24M	7% (4 - 10)	2% (1 - 4)	2% (1 - 4)	1% (0 - 3)
	30M	6% (4 - 10)	1% (0 - 3)	4% (3 - 6)	2% (1 - 4)
	36M	8% (6 - 11)	0	2% (1 - 4)	2% (1 - 4)
used in other location	6M	43% (26 - 61)	26% (15 - 41)	12% (7 - 19)	42% (29 - 55)
	12M	32% (23 - 42)	10% (6 - 15)	8% (5 - 12)	23% (17 - 31)
	24M	14% (11 - 19)	10% (7 - 14)	5% (4 - 7)	9% (7 - 12)
	30M	8% (6 - 12)	3% (1 - 5)	2% (0 - 3)	5% (3 - 8)
	36M	3% (2 - 5)	4% (2 - 6)	3% (1 - 4)	2% (1 -4)

Given away to relatives	6M	39% (23 - 58)	33% (20 - 49)	7% (3 - 14)	15% (8 - 27)
	12M	18% (11 - 27)	13% (8 - 19)	10% (6 - 14)	16% (10 - 22)
	24M	9% (6 - 13)	9% (7 - 13)	2% (1 - 4)	2% (1 - 4)
	30M	5% (3 - 7)	6% (4 - 9)	2% (1 - 4)	4% (2 - 6)
	36M	3% (2 - 5)	3% (1 - 5)	0	2% (1 - 4)
Destroyed accidentally	6M	0	2% (0 - 15)	5% (2 - 11)	4% (0 - 14)
	12M	3% (1 - 10)	3% (1 - 8)	5% (3 - 9)	11% (7 - 9)
	24M	3% (1 - 5)	7% (5 - 11)	2% (1 - 4)	11% (8 - 14)
	30M	2% (1 - 4)	6% (4 - 9)	2% (1 - 3)	5% (3 - 7)
	36M	1% (0 - 3)	4% (2 - 6)	2% (1 - 4)	2% (1 - 4)
Stolen	6M	4% (0 - 22)	0	2% (0 - 7)	4% (0 - 14)
	12M	3% (1 - 10)	2% (0 - 6)	1% (0 - 4)	1% (0 - 5)
	24M	2% (0 - 4)	2% (0 - 4)	1% (0 - 2)	2% (0 - 3)
	30M	3% (2 - 6)	2% (1 - 4)	3% (2 - 5)	1% (0 - 3)
	36M	3% (2 - 6)	2% (0 - 4)	3% (2 - 5)	0

Table S3a: Survivorship of the LLIN per time point

Net type	% Survival, 95%CI				
	6month	12month	24month	30month	36month
Interceptor®	93.7% [91 - 95]	84.0% [81 - 87]	59.4% [56 - 63]	47.2% [43 - 52]	37.1% [33 - 41]
Interceptor® G2	90.9% [88 - 93]	78.9% [76 - 82]	56.8% [53 - 60]	42.0% [38 - 46]	36.7% [33 - 41]
Olyset™ Plus	82.1% [79 - 85]	49.2% [46 -52]	18.1% [15 - 21]	14.8% [12 - 18]	9.5% [7 - 12]
Royal Guard®	89.9% [87 - 92]	70.1% [67 - 73]	39.9% [376 - 44]	27.4% [24 - 31]	18.0% [15 - 21]

Table S3b: Functional survival of the LLIN per time point

Net type	% function survival, 95%CI				
	6month	12month	24month	30month	36month
Interceptor®	91.7% [88 - 94]	76.7% [73 - 80]	44.3% [40 - 48]	30.5% [27 - 34]	21.8% [19 - 25]
Interceptor® G2	88.5% [85 - 91]	63.2% [59 - 67]	39.9% [36 - 44]	23.1% [20 - 27]	19.7% [16 - 23]
Olyset™ Plus	62.6% [58 - 67]	29.4% [26 - 33]	8.3% [6 - 11]	3.2% [2 - 5]	3.9% [3 - 6]
Royal Guard®	88.6% [85 - 91]	62.2% [58 - 66]	29.9% [26 - 34]	15.4% [13 - 19]	8.6% [7 - 11]

Figure S4: Proportion of nets with at least one hole per survey

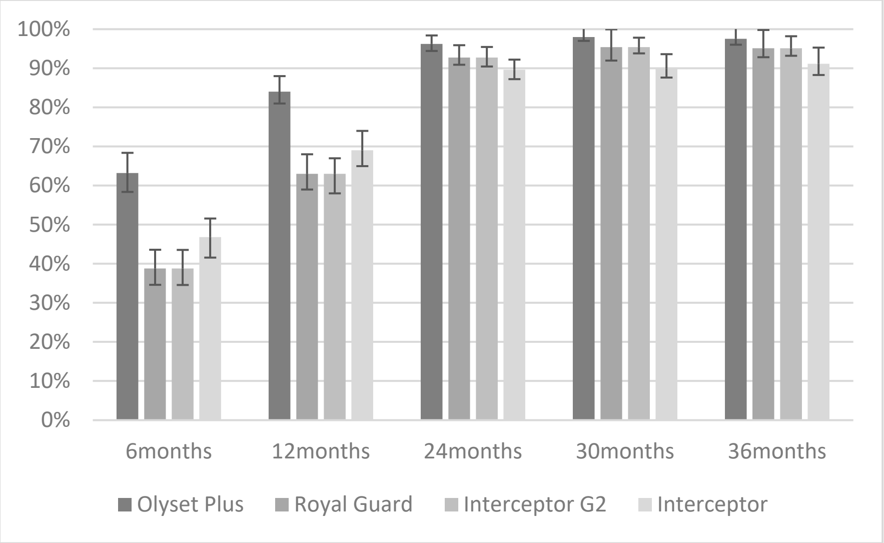

Malaria Prevention Trial: Misungwi (MRC/WT/DFID)

Household questionnaire Survey for Phase III study (English) v2.0 09/03/2019

Date (dd/mm/yy)	__ __/ __ __/ __ __		
Interviewer Initials	|_______|		
Identification			
Hamlet Code	|____|____|____|		
Ward name			
Village Name			
Hamlet Name			
Cluster Number	|_____|______|		

Household number/Unique address	|____|_____|____|_____|_____|_____|		

Interview Information		
Question description	Options	Answer
Consent result	Consent given	1
	Ineligible (no children under 15)	2
	Return later	3
	Refused	4
	Dwelling vacant for survey duration	5
	Dwelling not found	6
	Dwelling not visited	7
Total number of visits	|____|	
Comments		

Socio Economical Status						
No.	Question description	Options		Answer	If	Goto
9	Has the head of the household ever attended school?	Yes		1		
		No		0	0	11

		Don't Know		98	98	11
10	What is the highest level of school the head of the household attended:	Primary		1		
		Secondary/technic		2		
		Higher		3		
		Don't Know		98		
11	Has the head of the household WIFE ever attended school?	Yes		1		
		No		0	0	13
		Not Applicable		2	2	13
		Don't Know		98	98	13
12	What is the highest level of school the head of the household WIFE attended:	Primary		1		
		Secondary/technic		2		
		Higher		3		
		Don't Know		98		
13	How many rooms are there in this household?<br>>>Include all structures (huts etc)	Number (Don't Know = 98)		_ _		
14	How many rooms in this household are used for sleeping?	Rooms number		|_____|		
15	How many sleeping places are there in this household (beds, mattresses or mats)?<br>>>Ask for both inside the hut and outside	Number		_ _		
16	How many sleeping places were used last night?					
			Check box			18
17	What is the main material of the roof? (observe)	Grass/Papyrus/leaves		1		
		Metal sheets		2		

	(>>Multiple answers allowed)	Metal sheets	3		
		Other	4	4	17b
17b	If Other type of roof specify	Free text			
18	What is the main material of the floor? (>>Multiple answers allowed)	Earth/sand	1	1	19
		Cement	2	2	
		Tiles	3		
		Other	4	4	18b
18b	If Other type of floor specify	Free text			
19	What is the main material of the walls? (>>Multiple answers allowed)	Grass/leaves	1	1	20
		Mud	2		
		Burnt bricks	3		
		Unburnt bricks	4		
		Cement bricks	5		
		Other	6	7	19b
19b	If Other type of wall specify	Free text			
20	Are the walls plastered? (>>Multiple answers allowed)	Yes completely	1	1	
		Partially/damaged	2	2	
		No	0	0	
21	Are eaves open? (Is there a gap between the top of the wall and the roof?)	Yes	1	1	
		No	0	0	
22	Does the house have a ceiling?	Intact	1	1	
		Damage/Partial/traditional	2	2	
		No ceiling	0	0	
23	Where does the Households main income come from?	Fishing/Farming/Selling cash crops	1	1	24
		Mining	2	2	
		Buisness/Shop	3	3	
		Medical/Teacher/Goverment	4	4	

		Other		5	5	23b
23b	If Other kind of income specify	Free text				
24	Does the household (any member) have any of the following (Multiple choices)		Yes	No		
		Electricity	1	0		
		Radio	1	0		
		Mobile phone	1	0		
		Bicycle	1	0		
		Car, boat, motorbike	1	0		
		Sewing machine	1	0		
		Television	1	0		
		Livestock	1	0	1	25
25	Number of animals the household owns?<br>>> write 000 if none<br>if don't know write 9999<br>(Multiple choices)	Poultry/birds		_ _		
		Goats and sheep		_ _		
		Pigs		_ _		
		Cows/Donkeys		_ _		
		Other		_ _		25a
25a	Other specify	Free text				
25b	Are the goat/sheep or cows staying inside the house at night?	Yes		1		
		No		0		
		Don't Know		9999		
26	What is the main source of drinking water for members of your household? (choose only one)	Piped water				
		Piped into dwelling		1		
		Piped to yard/plot		2		
		Piped to neighbor		3		
		Public tap/stand pipe		4		
		Dug well				
		Protected well		5		

		Unprotected well	6		
		Water From Spring	7		
		Rainwater	8		
		Surface Water (River/Dam/Lake/Pond/Stream	9		
		Other, Specify________________________			
26b	If other source of drinking water, specify	Free text			
27	What kind of toilet facility do members of your household usually use? (observe)	Flush Toilet	1		
		Ventilated improved Pit Latrine	2		
		Traditional Pit Latrine	3		
		None/bush	4		
		Other, Specify______________________			
27b	If other kind of toilet, specify	Free text			
28	What type of fuel does your household mainly use for cooking?	Firewood/straw	1		
		Charcoal	2		
		LPG/Natural gas	3		
		Biogas	4		
		Electricity	6		
		Paraffin	7		
		Other, Specify______________________			
28b	If other fuel, specify	Free text			
29	Does the household own land used for farming?	Yes	1		
		No	0	0	30
30	If yes, indicate approximate size in acres >> if don't know write 9999	Acres	|_____|		
31	What time did you go inside your house last night?	Before 18 hours	1		

		18 -19 H	2
		19 -20 H	3
		20 -21 H	4
		21 -22 H	5
		After 22 H	6
		Don't know	9999
32	What time was the door closed for the night last night?	Before 18 hours	1
		20 -19 H	2
		21 - 20 H	3
		22 -21 H	4
		23 -22 H	5
		After 22 H	6
36	My family is at risk of malaria all-year round	Strongly Disagree	1
		Disagree	2
		Neither	3
		Agree	4
		Strongly Agree	5
37	It's more important to me to use nets in the rainy season than in the dry season	Strongly Disagree	6
		Disagree	7
		Neither	8
		Agree	9
		Strongly Agree	10
38	It makes me worried when one of my children might have malaria	Strongly Disagree	11
		Disagree	12
		Neither	13
		Agree	14

		Strongly Agree	15	
39	It makes me worried when me or my spouse get malaria	Strongly Disagree	16	
		Disagree	17	
		Neither	18	
		Agree	19	
		Strongly Agree	20	
40	I don't worry much about malaria these days	Strongly Disagree	21	
		Disagree	22	
		Neither	23	
		Agree	24	
		Strongly Agree	25	
41	The last time someone in this household was diagnosed with malaria was:	In the last 3 months	1	
		In the last 6 months	2	
		In the last 12 months	3	
		Over 12 months ago	4	
		Don't Know	9999	
42	The last time someone I know died of malaria was:	In the last 3 months	1	
		In the last 6 months	2	
		In the last 12 months	3	
		Over 12 months ago	4	
		Don't Know	9999	
43	How many mosquito nets that can be used for sleeping does your household have? (Probe for any nets currently not in	Number Nets _ _	Net >0	44
			Net=0 or DNK	###
		Don't know 98	98	

	use, stored, saved, still in packaging etc)		
Questions on each net Hole index			
No.	Question	Options	
	Mosquito net number	I_ _ _I	
44	Is the net an LLIN?	P8X01MS-LNBE	1
		58147523	2
		58574747	3
		1.CL88190.1.BL.08.18	4
		Olyset Net	5
		Permanet 2	6
		Olyset plus	7
		Other LLIN	8
		No it is not a LLIN	9
		Don't know	9999
45	How does the loop look like? (observe)	White piece of netting	1
		Blue piece of netting	2
		Blue ribbon	3
		Pink ribbon	4
46	How many month ago was this net obtained?<br>Enter "00" if less than one month	Months	
		Don't remember	9999
1	How many month ago did you start to use this net?<br>Enter "00" if less than one month	Months	
		Don't remember	9999

47	Was the net observed by the FW	Yes	1
		No	0
		Yes goto	48
48	How is the net found? (observe)	Hanging loose over sleeping place	1
		Hanging tied in knot	2
		Hanging folded	3
		Visible but not hung up	4
		Store away	5
49	What type of bed is the net hanging over? (observe)	Wooden or iron bedframe (improved)	1
		Stick bedframe	2
		No bedframe	3
		Other, specify	4
49b	If the response to "what type of bed is the net hanging over" is other specify	Text	
50	What type of mattress/sleeping material is used with this net? (observe)	No mattress	1
		Reed mat (mkeka)	2
		Clothes/other net/material	3
		Foam/spring mattress	4
		Hammock	5
		Other, specify	6
50b	If the response to "what type of bed is the net hanging over" is other specify	Text	
51	Where was this net obtained	PAMVERC Project campaign	1
		School Net Program	2
		Antenatal clinic when pregnant	3
		Universal campaign 2015	4
		Other faith-based programme or NGO	5

		Purchased full price	6
		Received for free by friend/family	7
		Don’t Know	9999
52	How many nights has this net been used in the last week (the last 7 days)?	Every night (7 nights)	1
		Most nights (5-6)	2
		Some nights (1-4)	3
		Not used last week	4
		Net is not used at all	5
		Don’t know	9999
		if net not used last week or at all go	
53	Was this net used by any person last night?	Yes	1
		No	0
		Don’t Know	9999
		No goto	57
54	Who used this net last night?<br><br>>> probe for any additional person using this net last night and enter line number from Q01	Resident number of users (Q01)	
		|_____|_____|	1
		|_____|_____|	2
		|_____|_____|	3
		|_____|_____|	4
		|_____|_____|	5
		|_____|_____|	6
56	How long has the net been in use?	1 week or less	1
		1 month or less	2
		1 to 3 months	3
		More than 3 months	4
		Don't know	9999

57	Why were the nets not used	I can't use it answer	
		No enough space	1
		No sleeping place to cover	2
		Being washed	3
		Usual user(s) did not sleep here	4
		Don't want to use it answers	
		No mosquitoes now	5
		Too hot	6
		Net too old or torn	7
		Net too dirty	8
		Presence of bedbugs	9
		Reserved for future use	10
		Net not hung	11
		Used for other purpose	12
		Don't know	9999
		Other	13
		If Other goto	57b
57b	If, other specify	Text	
58	How does this current net compare to the net you had previously	This net is better	1
		This net is worse	2
		The net is the same	3
		Don't know	9999
59	If this net is better, why?	Less/fewer can penetrate net	1
		The net is soft (polyester)	2
		Does not acquire holes easily	3
		Other	4
		Don't know	9999

60	If this net is worse, why?	Much mosquitoes penetrate through the net	1
		The net is smaller	2
		The net is hard (polyethylene)	3
		Net acquire holes easily	4
		The net has a bad odor	5
		This net causes some side effects	6
		Other	7
		Don’t know	9999
61	Do you think this net is still protective?	Yes	1
		No	2
		Don't know	9999
62	During which periods of the year is this net used to sleep under?	All year	1
		Only the rainy season	2
		Only the dry season	3
		Don't know	9999
63	Do you tuck the net in at night?.	Yes	1
		No	2
		Don't know	9999
64	Has the net ever been washed?	Yes	1
		No	2
		Don't know	9999
		Yes goto	66
65	How many times was the net being washed	1-5 time	1
		5-10 times	2
		10-15 times	3
		15-20 times	4
65b	If others specify	Text	

66	When was the last time you washed the net	less than 1 week ago	1
		1 week to 1 month ago	2
		1-3 months ago	3
		3-6 months ago	4
		> 6 months ago	5
		Don't know	9999
67	Do you use an open flame for cooking, heating or lighting where the net is found?	Yes	1
		No	0
		Don't Know	9999
		Yes goto	68
68	If yes which type of open flame are you using	Wood fire	1
		Charcoal fire	2
		Wax candle	3
		Oil lamp with a glass	4
		Oil lamp without a glass	5
		Other, specify	6
69	Was the net selected to count the hole	Yes	1
		No	0
		No GO TO NEXT NET or Resident section	
70	Does the net has any hole	Yes	1
		No	0
		No GO TO NEXT NET or Resident section2 or mosquito number in second day	
72	ZONE 1 (TOP PANNEL) Number of holes size 1	Not larger than a finger	☐☐

73	ZONE 1 (TOP PANNEL) Number of holes size 2	Larger than finger but not larger than hand width	☐☐
74	ZONE 1 (TOP PANNEL) Number of holes size 3	Larger than hand width but smaller than head	☐☐
75	ZONE 1 (TOP PANNEL) Number of holes size 4	Larger than head	☐☐
76	ZONE 2 Number of holes size 1	Not larger than a finger	☐☐
77	ZONE 2 Number of holes size 2	Larger than finger but not larger than hand width	☐☐
78	ZONE 2 Number of holes size 3	Larger than hand width but smaller than head	☐☐
79	ZONE 2 Number of holes size 4	Larger than head	☐☐

80	ZONE 3 Number of holes size 1	Not larger than a finger	☐☐
81	ZONE 3 Number of holes size 2	Larger than finger but not larger than hand width	☐☐
82	ZONE 3 Number of holes size 3	Larger than hand width but smaller than head	☐☐
83	ZONE 3 Number of holes size 4	Larger than head	☐☐
84	ZONE 4 (Bottom pannel) Number of holes size 1	Not larger than a finger	☐☐
85	ZONE 4 (Bottom pannel) Number of holes size 2	Larger than finger but not larger than hand width	☐☐
86	ZONE 4 (Bottom pannel) Number of holes size 3	Larger than hand width but smaller than head	☐☐

87	ZONE 4 (Bottom pannel) Number of holes size 4	Larger than head	
88	ROOF Number of holes size 1	Not larger than a finger	
89	ROOF Number of holes size 2	Larger than finger but not larger than hand width	
90	ROOF Number of holes size 3	Larger than hand width but smaller than head	
91	ROOF Number of holes size 4	Larger than head	
92	What types of hole are observed?	Horizontal tears at bottom	1
		Holes at hanging points	2
		Open seams	3
		Burn holes	4
		Holes from rodents	5
		Whole section missing	6
93	Number of holes repaired	Total	

94	Have you tried to fix any holes in the net	Yes	1
		No	2
		Don't know	9999
		IF No GO TO	96
95	How did you repair the hole?	Stitched	1
		Knotted/tied	2
		Patched	3
		Other way, specify	4
95b	If the response to "How did you repair the hole" is other way specify	Text	
96	If not, what was the main reason?	Too busy/no time	1
		Not necessary, the net is still good	2
		Don't know how to fix	3
		Too damaged to fix	4
		Other, specify	5
96b	If the response to "If not, what was the main reason" is other way specify	Text	
